# Nanotechnologies for the detection and treatment of endometriosis

**DOI:** 10.3389/fbiom.2023.1279358

**Published:** 2023-11-16

**Authors:** Maneesha Sahni, Emily S. Day

**Affiliations:** 1 Department of Biomedical Engineering, University of Delaware, Newark, DE, United States,; 2 Department of Materials Science and Engineering, University of Delaware, Newark, DE, United States,; 3 Center for Translational Cancer Research, Helen F. Graham Cancer Center and Research Institute, Newark, DE, United States

**Keywords:** women’s health, reproductive health, nanoparticles, nanomedicine, diagnostics, therapeutics, gynecologic health

## Abstract

Endometriosis is an incurable gynecologic disease characterized by endometrial-like tissue growth outside of the uterine cavity. It affects approximately 10% of reproductive age women, who endure pelvic pain during periods and/or sexual intercourse and who suffer from reduced fertility and diminished quality of life due to the side effects of current treatments. To improve the management and prognosis of endometriosis patients, researchers have recently begun to develop nanoparticle-based diagnostics and treatments that are more effective and less invasive than existing approaches. This review discusses the current state of the field and highlights considerations for the continued development of nanotechnologies for the diagnosis and treatment of endometriosis.

## 1 Introduction

**FIGURE 1:**
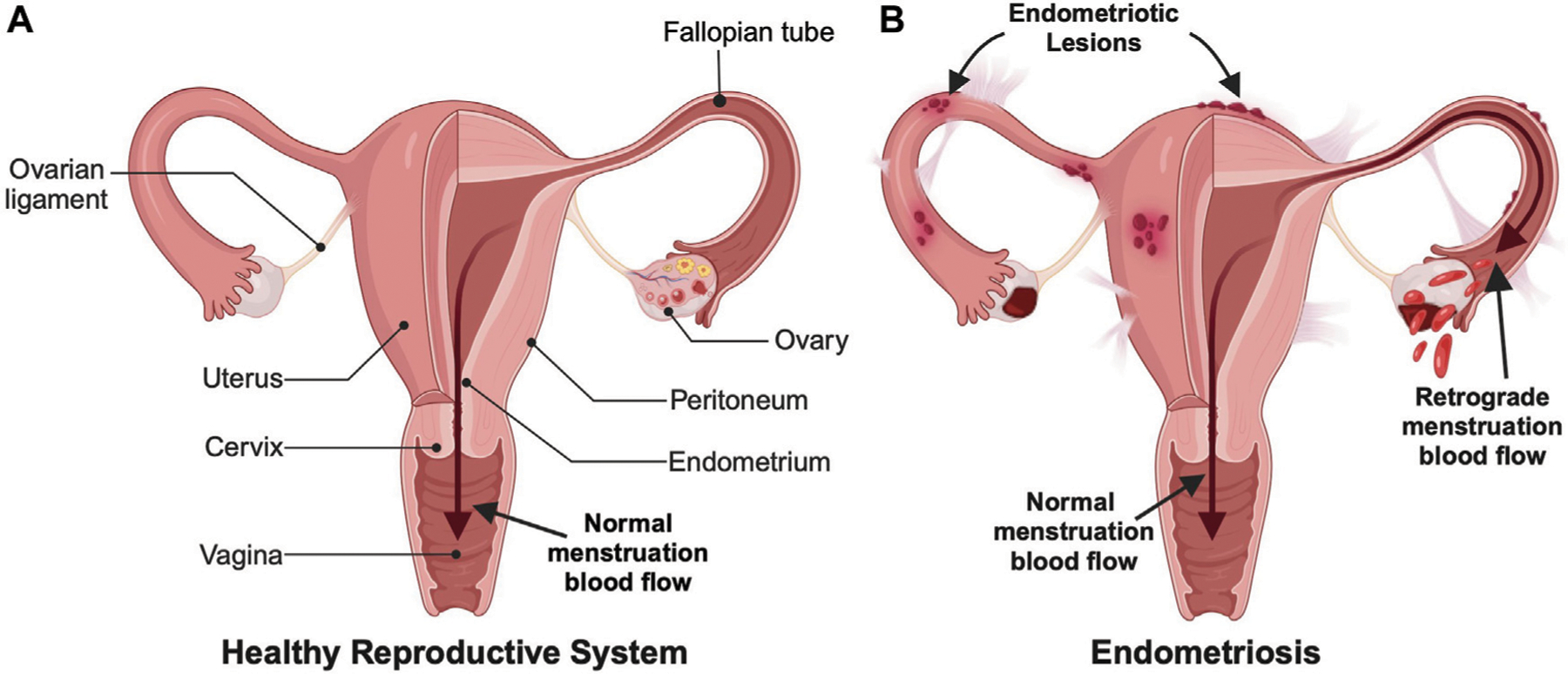
Comparison of (**A**) a healthy female reproductive system and (**B**) an endometriotic reproductive system. Endometriosis involves both endometrial lesions that form outside the uterine cavity and retrograde menstruation, which are commonly used for diagnosis. (Created with BioRender.com).

Approximately 10%–15% of reproductive-aged women and girls around the world are diagnosed with endometriosis (Eisenberg et al., 2018). Endometriosis is an inflammatory disease where endometrium-like tissue, which usually lines the inside of the uterus in a healthy patient, grows outside of the uterus where it can spread to other organs including the ovaries, fallopian tubes, bladder, and bowel (Figure 1) (Ellis et al., 2022). This abnormal tissue growth develops into cysts, known as endometriotic lesions, as well as scar tissue. In a eutopic, or healthy, endometrial lining, endometrial cells provide the pathway for a fertilized embryo to attach during the onset of pregnancy (Reed et al., 2018; Liu and Lang, 2011). During a standard menstrual cycle, the hormones produced by the ovaries stimulate these cells, which causes them to grow and divide. In the absence of fertilization/pregnancy, the endometrial cells die and are shed and exit through the vagina, which is the process commonly known as the menstrual cycle or period. In endometriosis patients, the endometriotic lesions in heterotopic tissues contain the same endometrial cells, which respond to the ovarian hormones the same way they would respond in a eutopic uterus. This causes the lesions to thicken and swell with blood at specific points in the menstrual cycle, which causes inflammation, pain, and discomfort. Symptomatic women commonly experience pain during menstruation, sexual intercourse, urination and/or bowel movements (Ellis et al., 2022). Unfortunately, accurately diagnosing endometriosis based on these symptoms alone remains a clinical challenge.

It is unknown why some women develop endometriosis and others do not, but there are multiple factors and theories behind what contributes to endometriosis onset and development. One possible cause is retrograde menstruation, which is when menstrual blood travels back through the fallopian tubes instead of outward through the cervix and vagina (Figure 1) (Agostinis et al., 2021). This backflow of blood can result in the implantation and growth of endometrial cells outside the uterus. Another possible cause could be cellular metaplasia, which is when cells outside the uterus differentiate into endometrial-like cells due to an environmental stimulus such as hormones or immunological factors (Giroux and Rustgi, 2017). For example, some studies suggest increased estrogen levels could be an inducing factor for endometriosis. Finally, certain hereditary and/or physical factors can increase the risk of a woman developing endometriosis, such as having a mother, sister, or daughter with endometriosis, having an abnormal uterus, starting menstruation before the age of 11, and having either shorter menstrual cycles that are less than 27 days or heavy and long menstrual periods lasting more than 7 days (Tsamantioti and Heba, 2023).

There is a need for noninvasive diagnostics and a cure for endometriosis. Current treatment options provide temporary relief, such as pain management through the intake of anti-inflammatory medications like ibuprofen (Tsamantioti and Heba, 2023). Or, if hormone imbalance is involved, women may be prescribed hormonal treatments such as oral contraceptives. Another form of treatment is surgically excising the endometriotic lesions, which can help alleviate pain and discomfort and return affected organs back to their original state. Surgical excision of endometriotic lesions can also improve women’s fertility by removing the source of inflammation that affected the functionality of the ovaries, fallopian tubes, and/or uterus (Ellis et al., 2022). Additionally, surgery can overcome endometriosis-induced distortion or blockage of the fallopian tubes, which limits their ability to capture the egg after ovulation, thus contributing to infertility. In some instances, surgical excision is combined with ablation to destroy the lining of the uterus, which reduces the heavy menstrual bleeding associated with endometriosis. Ablation methods include electrical ablation, radiofrequency ablation, cryoablation, hydrothermal ablation, microwave ablation, and balloon therapy. While effective, endometrial ablation may not be suitable for women who wish to become pregnant because it increases risk to both the mother and baby. Beyond surgical excision of lesions to improve fertility, other noninvasive treatments for infertility caused by endometriosis include oral or injectable medicines to stimulate super-ovulation, insemination, and *in vitro* fertilization (Lee et al., 2020). Despite the benefits of surgical treatment of endometriosis, complications can occur during or after the surgery and lesions can reoccur, with about 27% of patients needing multiple surgical procedures (Park et al., 2022). Hence, improved treatment methods are still required.

In terms of diagnostics, the two main techniques currently used by healthcare providers to confirm a diagnosis of endometriosis are a surgical procedure known as laparoscopy or advanced imaging modalities. In laparoscopy, a surgeon inflates the abdomen using a harmless gas, creates a small incision in the abdomen, and then uses a laparoscope to inspect the reproductive organs, intestines, and other nearby areas to identify any signs of endometriosis. Alternatively, imaging techniques including ultrasound and magnetic resonance imaging (MRI) can be utilized to try to noninvasively detect endometriosis, but these are limited by cost and lack the needed resolution for accurate identification of endometriotic lesions (Tsamantioti and Heba, 2023).

Given the limitations of current diagnostic and treatment methods, researchers and clinicians are searching for new ways to better manage endometriosis. Previously, nanoparticle-based contrast agents and drug carriers have shown great promise in improving the diagnosis and treatment of various diseases. Now, researchers are interested in applying these tools to applications in women’s health (Lloyd-Parry et al., 2018; Nelson et al., 2021; Swingle et al., 2023). One challenge in the development of new diagnostics and therapeutics for endometriosis is the paucity of preclinical animal models that accurately represent the human disease. Most studies rely on rodent models wherein endometriotic cells or donor uterine tissue are transplanted subcutaneously or intraperitoneally into recipient animals to induce disease. An alternative model is macaques which can naturally develop the disease, but these are more expensive and less readily accessible. Despite their drawbacks, these models have proven useful in the preclinical evaluation of nanoparticle-based tools to manage endometriosis. In the following sections, recent preclinical studies into the use of nanotechnologies for the diagnosis and treatment of endometriosis are discussed.

## 2 Diagnostics

This section discusses the development of nanotechnologies to noninvasively diagnose endometriosis and determine its level of severity. Section 2.1 introduces nanotechnologies for biomarker detection, while Section 2.2 describes nanoparticle contrast agents for imaging of endometriosis lesions.

### 2.1 Nanotechnologies as biosensors for endometriosis biomarker detection

The detection of biomarkers, including proteins and/or genetic material, is an appealing non-invasive approach to diagnose endometriosis. Previous studies have shown that endometriosis patients have a distinct microbial makeup in their peritoneal fluid and feces when compared to healthy patients (Ellis et al., 2022). Based on these observations, *Ruminococcus* in the gut and *Pseudomonas* in the peritoneal fluid may serve as diagnostic signatures for endometriosis. Additionally, the follicular fluid of endometriosis patients has dysregulated cytokine profiles with significant upregulation of IL-1β and IL-6 and lower concentrations of anti-inflammatory cytokine IL-12 and inflammatory cytokine IL-10, as well as lower expression of the cell adhesion molecule E-cadherin (Ellis et al., 2022). Finally, endometriosis patients have abnormally elevated levels of Cancer Antigen 125 (CA125) in serum (above 35 units per mL), so the CA125 assay is currently utilized to diagnose endometriosis (Kalyani et al., 2021). Recently however, CA19–9 has emerged as a more promising biomarker compared to CA125 due to its improved sensitivity and specificity (Kurdoglu et al., 2009; Hsu et al., 2010). In the future, nanotechnology-based biosensors that detect the presence or absence of such biomarkers in patient samples derived from blood, urine, feces, or other fluids could provide more accurate and less invasive diagnosis of endometriosis than existing surgical or imaging methods.

Before introducing specific examples of nanotechnology-based biosensors for endometriosis, it is important to understand the basic concept and benefits of these tools *versus* traditional sensors. Typically, biosensor assays use a reporter (such as an antibody tagged with a fluorophore) to detect the presence of a specific antigen (such as CA19–9 in serum). Nanoparticle-based systems can increase the sensitivity and signal-to-noise ratio of these assays by either increasing the readout (e.g., elevating the fluorescence quantum yield) or increasing the strength of binding to the targeted molecule. This extends into other detection methods as well, including those based on absorbance, electrical measurements, and more. A few examples of electrochemical immunosensors that have been reported to date are described below.

One team developed a bio-nanocomposite to detect CA19–9 in serum. The sensor consisted of multiwalled carbon nanotube (MWCNT) and magnetite (Fe_3_O_4_) nanoparticles dispersed in chitosan (CS), which were coupled to anti-CA19–9 antibodies and then placed onto a glassy carbon electrode using glutaraldehyde as a cross-linker (Kalyani et al., 2021). The electrical signals were recorded upon addition of serum containing CA19–9 antigen using square wave voltammetry in the presence of redox marker [Fe (CN)_6_]^3−/4−^ solution, and the sensing was validated using ELISA (enzyme linked immunosorbent assay) techniques. This sensor could detect CA19–9 across a range of 0.00083 to 83 IU/mL, which spans most of the physiological range (one study reported the mean serum CA19–9 level in endometriosis patients is ~15 IU/mL for stage 1 disease and ~108 IU/mL for stage IV disease (Harada et al., 2002). Other studies have shown supportive data for the elevated levels of CA19–9 in serum, especially in advanced stages of the disease, with values ranging from 16.81 IU/mL for stage I to 111.66 IU/mL for stage IV (Tuten et al., 2014; Kalyani et al., 2021). Accordingly, this test has potential application in early-stage diagnosis and/or treatment monitoring.

**FIGURE 2:**
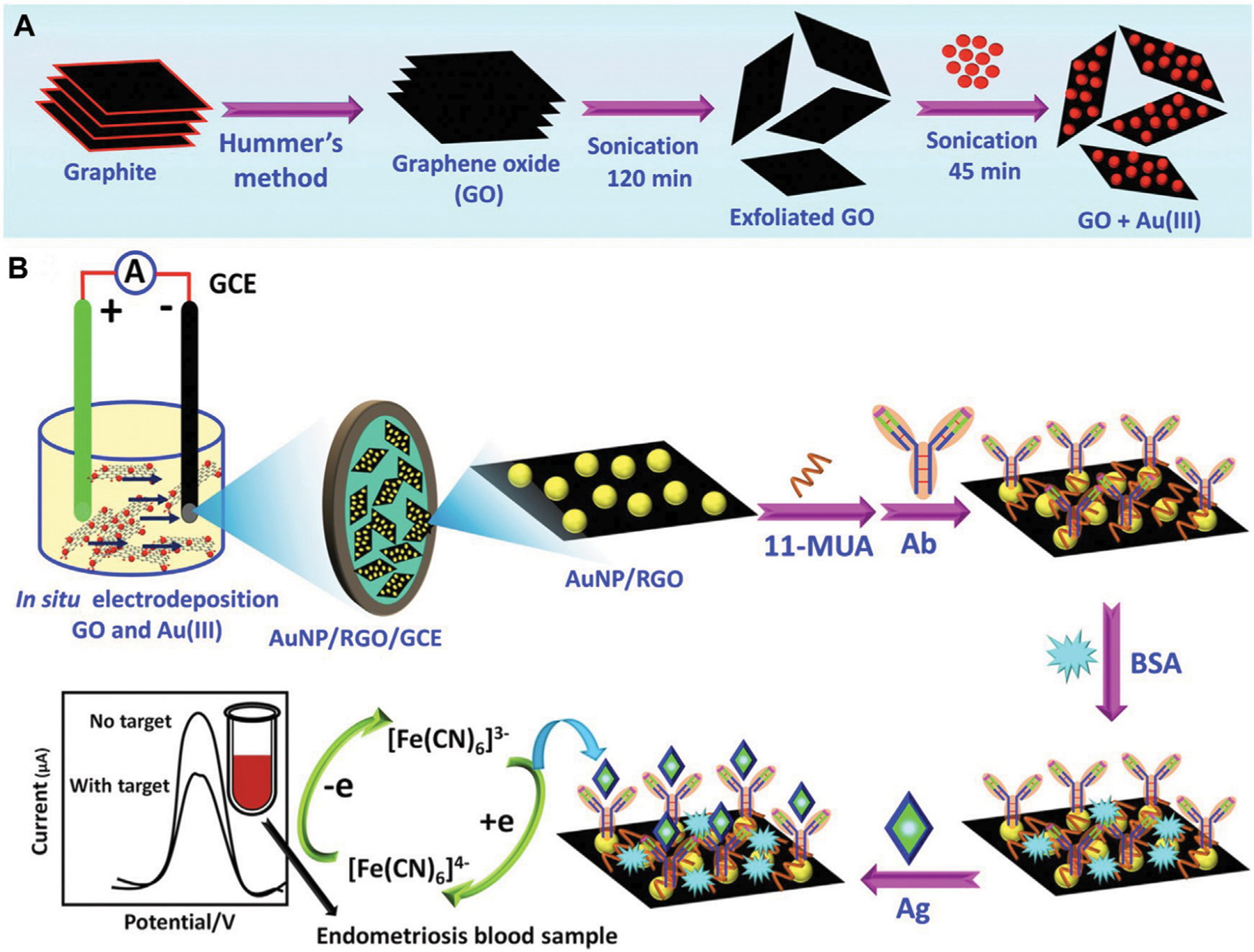
Overview of a nanocomposite designed to detect CA-125 in patient samples. (**A**) First, graphene oxide (GO) and Au^3+^(AuIII) composites were prepared as depicted. (**B**) Prepared GO + AuIII composites were used to make glassy carbon electrodes (GCE) modified with AuNP/reduced graphene oxide (AuNP/RGO/GCE). Next, 11-mercaptoundecanoic acid (11-MUA) was used to facilitate attachment of anti-CA125 antibodies. After blocking nonspecific active sites with bovine serum albumin (BSA), CA-125 antigens (Ag) (diluted in PBS or within patient blood samples) were added to the sensors and square wave voltammetry analysis was implemented to quantify CA-125. Adapted with permission from Sangili A, *et al. ACS Applied Bio Materials.* 2020; 3 (11): 7620–30. Copyright 2020, American Chemical Society.

Gold nanoparticles (AuNPs) are also attractive for use in immunosensors due to their speed of synthesis, high conductivity, and ease of antibody labeling for biomolecule detection. Graphene is another material with advantageous properties for immunosensing applications including exceptional mechanical strength, large surface area, and high electrical conductivity. When reduced to graphene oxide (GO), the surface functional groups of GO interact with metal nanoparticles including AuNPs, allowing for the production of effective electrochemical sensors based on these nanocomposites. Sangili et al. developed a label-free immunosensor wherein glassy carbon electrodes (GCE) were modified with AuNP/reduced-GO, then further modified with anti-CA125 antibodies using a layer-by-layer assembly process (Figure 2) (Sangili et al., 2020). Bovine serum albumin (BSA) was used to block nonspecific active sites, then samples containing CA-125 (either phosphate buffered saline (PBS) with CA-125 at known concentrations or patient blood samples) were added for 45 min at 37°C. Thereafter, the analyte was detected using square wave voltammetry techniques. This sensor could detect CA-125 across a range of 0.0001–300 IU/mL, with a limit of detection (LOD) of 0.000042 IU/mL, and it showed excellent correlation with ELISA validation techniques when evaluated using patient samples. Moreover, the sensor had a broader detection range and lower LOD than previous immunosensors and the standard ELISA method. Hence, there is great promise for use of this and other immunosensors and biomarkers in the clinical screening of endometriosis. In the future, such low-cost, point-of-care diagnostic tools could improve patient access to screening.

### 2.2 Imaging techniques

Beyond biomarker detection, imaging techniques can be employed to diagnose endometriosis through lesion visualization. As mentioned earlier, current clinical imaging techniques include MRI and ultrasound. These and other imaging modalities can assist researchers and clinicians in learning more about the etiology and pathogenesis of the disease. Nanoparticle contrast agents that are coated with ligands that facilitate binding to endometrial cells or associated reproductive tissue structures can be used to enhance and improve these imaging techniques by providing more sensitive and accurate visualization and localization of the disease (Ellis et al., 2022). Some nanoparticles, such as those based on iron oxide or gold, have inherent properties that allow them to enhance the contrast of magnetic or optical imaging techniques. Other systems encapsulate molecules such as fluorescent dyes to achieve contrast enhancement between diseased and healthy tissues/cells. A key advantage of nanoparticle-enhanced imaging techniques is that they require a simple injection or infusion of the material, followed by non-invasive imaging of the tissues of interest. This is advantageous *versus* invasive laparoscopy techniques but requires effective delivery of the contrast agent to the targeted lesions. The main limitation of nanoparticle contrast agents is that they may be retained in the body with unknown long-term consequences. Clinicians and patients must carefully consider both the benefits and potential risks of nanoparticle contrast agents when deciding whether to use these materials for lesion detection.

**FIGURE 3:**
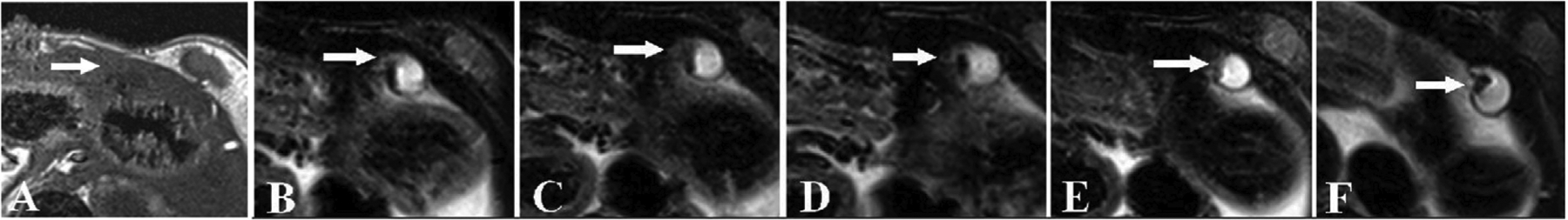
MRI images of rat ectopic endometriotic lesions at various time points post IV injection of HA-modified-Fe_3_O_4_ nanoparticles. (**A**) T1 weighted image of ectopic uterine tissue (EUT) (indicated by white arrow), which appeared as an ill-defined cystic mass with low signal. (**B**) Fat-suppressed (FS)-T2 weighted image of EUTs before nanoparticle injection. The EUTs showed a slightly higher signal intensity surrounded by the fibrous walls’ intermediate signals. In FS-T2 weighted images at (**C**) 15 min, (**D**) 30 min, (**E**) 60 min, and (**F**) 120 min post nanoparticle injection the lesion walls and lesion to background contrast are more visibly identifiable. Reproduced with permission from Zhang H, *et al. PLOS ONE.* 2014; 9 (4): e94718. Copyright 2014, PLOS ONE.

In a recent study, scientists developed magnetic iron oxide (Fe_3_O_4_) nanoparticles, which have high relaxivity, low toxicity, and exceptional contrast enhancement, as negative contrast agents for T2-weighted MRI of endometriosis (Zhang et al., 2014). In this work, the Fe_3_O_4_ nanoparticles were modified with hyaluronic acid (HA) to facilitate targeting of CD44 receptors that are overexpressed on endometriotic cells (Zhang et al., 2014). Endometriosis was surgically induced in rats by autologous transplantation. Four weeks post-surgery, rats received intravenous (IV) HA-Fe_3_O_4_ nanoparticles, then MRI was performed. By 2 h post-injection, there was significant darkening of the walls of the lesions in mice that received HA-Fe_3_O_4_ nanoparticles and were imaged using turbo spin-echo fat-suppressed T2-weighted MRI (Figure 3). The presence of the nanoparticles in the lesions was confirmed by quantifying Fe concentration and by staining excised tissues with Prussian blue. More nanoparticles were found in the ectopic endometrium as compared to eutopic endometrium, validating the use of CD44 as a marker for lesion targeting (Zhang et al., 2014).

Related to this work, Lee et al. reported the use of ultrasmall superparamagnetic iron oxide nanoparticles to detect intraperitoneal endometriosis lesions in an experimental rat model by MRI (Lee et al., 2012). These could detect lesions 3 mm or greater in size. More recently, Park et al. developed hexagonal iron oxide nanoparticles coated with a peptide targeting vascular endothelial growth factor receptor-2 (VEGFR-2) for the targeted imaging and treatment of endometriosis (Park et al., 2022). Studies performed in mice bearing transplants of macaque endometriotic tissue showed that, following IV administration, the nanoparticles could enhance both T2-weighted MRI and fluorescence contrast of endometriosis lesions (Park et al., 2022). The therapeutic uses of these nanoparticles will be discussed in Section 3, but the results point toward the exciting potential of nanotechnology-based platforms to improve the detection and treatment of endometriosis.

**FIGURE 4:**
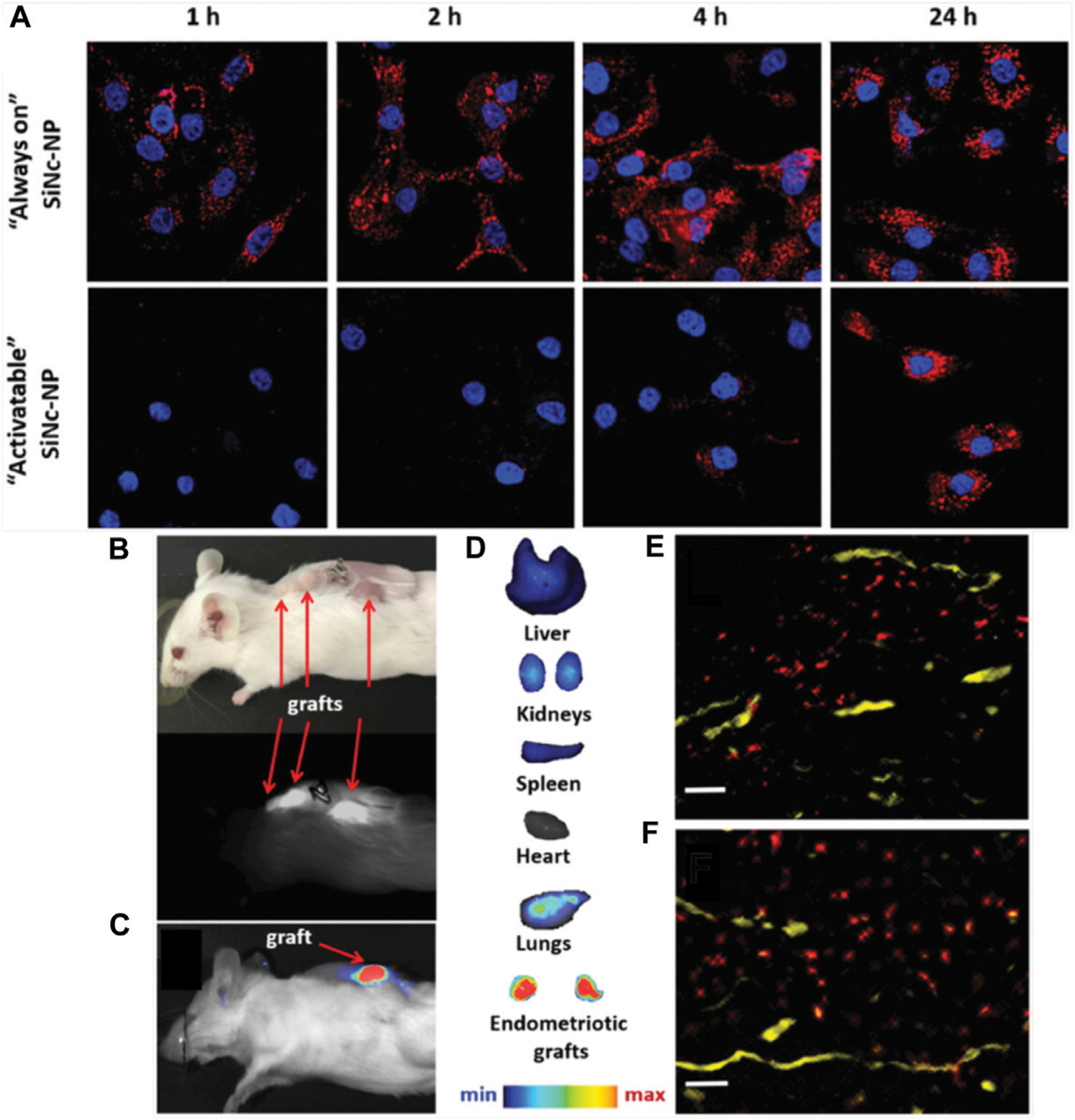
Detection of endometriosis with activatable SiNc-PEG-PCL nanoparticles. (**A**) Fluorescence microscopy images of macaque endometriotic stromal cells incubated with “always on” SiNc-NP (top row) or “activatable” SiNc-NP (bottom row) for 1, 2, 4, and 24 h. The red and blue colors reflect the fluorescence signal generated by SiNc and NucBlue (nuclei staining), respectively. (**B**) Photograph (top) and NIR fluorescence image (bottom), recorded with Fluobeam 800, of a mouse bearing endometriotic grafts 24 h after IV injection of “activatable” SiNc-NP. (**C, D**) NIR fluorescence images of a mouse bearing endometriotic graft (**C**) and excised tissues (**D**) recorded with Pearl Impulse Small Animal Imaging System 24 h after IV injection of “activatable” SiNc-NP. (**E, F**) Fluorescence microscopy images of sections of endometriotic grafts collected 24 h after IV injection of SiNc-NP. Red indicates NIR fluorescence generated by SiNc-NP. Yellow represents blood vessels stained with fluorescently labeled anti-CD31 antibodies. Scale bars = 50 μm. Adapted with permission from Moses A, *et al. Small.* 2020; 16 (18): 1906936. Copyright 2020, Wiley VCH.

Finally, one study investigated the use of silicon naphthalocyanine loaded poly (ethylene glycol)-poly(ε-caprolactone) (SiNc-PEG-PCL) nanoparticles for fluorescence imaging of endometriosis (Moses et al., 2020). SiNc was selected as the contrast agent because it has greater fluorescence intensity and photostability than other dyes like indocyanine green. When encapsulated, the SiNc fluorescence was quenched, but upon nanoparticle uptake by endometrial cells, the SiNc molecules were released, activating their fluorescence and providing high signal-to-noise ratio (Figure 4A). *In vivo* studies were performed using an FDA-approved intraoperative imaging system, demonstrating the potential for nanoparticle-based contrast agents to enhance surgical detection and/or excision of endometriotic lesions (Figures 4B–F) (Moses et al., 2020). This system was also utilized for photothermal ablation of lesions, as discussed in Section 3. Compared to MRI, fluorescence imaging has reduced depth of penetration, but the fast acquisition, ease of use, and ability to be incorporated into routine surgical procedures are major benefits towards clinical implementation.

## 3 Treatment

Surgical ablation of endometriotic lesions via laparoscopy is the primary treatment option for patients with endometriosis. However, this treatment is invasive and incomplete removal of diseased tissue could lead to free endometriotic cells that can develop into recurrent lesions. Nanoparticles loaded with therapeutic agents and modified with targeting molecules can enable precise and effective treatment of endometriosis to avoid recurrence while also minimizing systemic side effects. The types of therapeutic agents that can be delivered with nanoparticles to treat endometriosis include anti-inflammatory drugs, hormonal therapies, and immunomodulating molecules. Alternatively, nanoparticles that produce heat when activated with light or an applied magnetic field can thermally ablate endometriosis lesions. This approach is less invasive than surgical ablation and may improve patient outcomes by yielding immunogenic cell death that minimizes recurrence. Each of these therapeutic strategies show promise to improve patient care.

### 3.1 Nanoparticle-enhanced photothermal therapy

Nanoparticle-enhanced photothermal therapy (PTT) is a treatment wherein photoresponsive nanoparticles are administered into the body and once they have accumulated at the disease site a near-infrared (NIR) laser tuned to the nanoparticles’ peak resonance wavelength is applied to the region, which causes the temperature of the targeted tissue to increase above 42°C as the particles convert the light energy into heat. PTT has been successfully applied to the treatment of solid-tumor cancers in both preclinical and human clinical studies (Riley et al., 2017; Rastinehad et al., 2019), and now researchers are investigating its potential to manage endometriosis (Giroux and Rustgi, 2017; Moses et al., 2020). A key benefit of PTT over drug-based therapies is that treatment is confined to areas where both the nanoparticles are laser light are combined—accordingly, the off-target effects of PTT are extremely low. Additionally, cells treated with heat are less prone to development of resistance, which results in improved overall response. Below, specific examples of nanoparticles developed for PTT of endometriosis are discussed.

Section 2 introduced the use of SiNc-PEG-PCL nanoparticles as fluorescence probes for endometriosis detection (Moses et al., 2020). Excitingly, these materials can also generate heat when exposed to NIR light, allowing their use for combination imaging and PTT of endometriosis. Both *in vivo* and *in vitro* testing were conducted to determine the efficacy of PTT with this system. When macaque endometriotic stromal cells were incubated with 30 μg/mL SiNc-NPs for 48 h and then irradiated with 780 nm light (0.9 W/cm^2^, 15 min), the temperature of the cells rose to ~53°C, resulting in >95% cell death (Moses et al., 2020). In the absence of irradiation, the cells remained viable, demonstrating the system’s cytocompatibility. *In vivo* studies were performed using mice bearing four endometriosis grafts each. The mice were intravenously injected with 3 mg SiNc/kg, and 24 h later two grafts per mouse were irradiated (780 nm light, 0.9 W/cm^2^, 15 min). Irradiated lesions experienced temperature rises to 47°C and were completely eradicated within 4 days after a single treatment with no recurrence during the study’s 7-week timeframe (Moses et al., 2020). These data indicate the SiNc system holds promise for the combined imaging and treatment of endometriosis.

**FIGURE 5:**
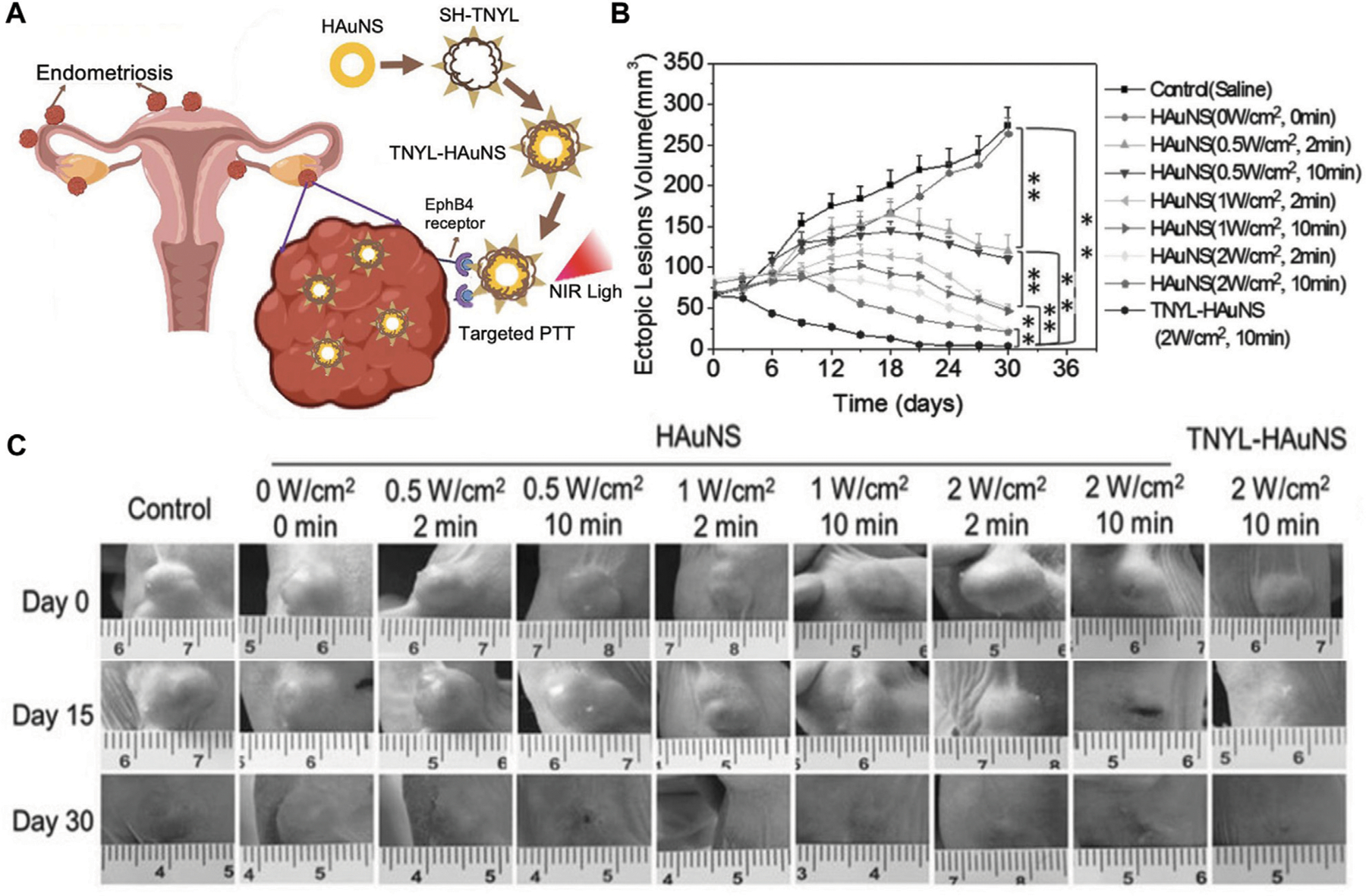
Photothermal therapy of endometriosis mediated by TNYL-targeted HAuNS. (**A**) Scheme depicting the ability of TNYL-HAuNS to bind EpHB4 receptors that are overexpressed in endometriosis lesions. Irradiation of the bound nanoparticles with NIR light facilitates photothermal ablation of the lesions. (**B**) Lesion growth curves for various treatment groups monitored every 6 days for 36 days. The TNYL-HAuNS treatment combined with 2 W/cm^2^, 10 min irradiation with a 780 nm laser significantly inhibited lesion growth (*n* = 6/group). (**C**) Representative images of lesions in mice from each treatment group at days 0, 15, and 30 post irradiation. (**A**) Adapted with permission Luo L, *et al. Small*, 2023; 2207694, Copyright 2023 Wiley VCH and (**B, C**) adapted with permission from Guo X, *et al. Small,* 2017; 13 (15): 1603270, Copyright 2017 Wiley VCH.

Similar promising results were obtained by Guo *et al.*, who developed a system for targeted PTT of endometriosis based on hollow gold nanoshells (HAuNS) coated with TNYL peptides that bind EphB4 receptors that are overexpressed in endometriosis lesions (Guo et al., 2017) (Figure 5A). When non-targeted HAuNS or TNYL-HAuNS were intravenously administered into mice bearing bilaterial endometriosis lesions, the TNYL-HAuNS displayed nearly 2-fold higher accumulation in the lesions based on inductively coupled plasma-mass spectrometry (ICP-MS) analysis of gold content in the tissues. In follow-up experiments, mice with endometriosis lesions received intravenous saline, HAuNS, or TNYL-HAuNS, followed by NIR irradiation of the lesions at levels ranging from 0 to 2 W/cm^2^ and times ranging from 0 to 10 min. A dosage-dependent response of the lesion volume was observed (Figures 5B, C), with the most effective treatment being TNYL-HAuNS combined with 2 W/cm^2^, 10 min irradiation. This combination inhibited lesion volume by 92.7%, compared to 77.2% for HAuNS under the same irradiation conditions. Saline combined with laser had minimal effect, demonstrating that PTT was mediated by the targeted HAuNS and not by the light alone (Guo et al., 2017). Overall, this study, combined with that of Moses et al., demonstrates that PTT presents an effective and safe treatment for endometriosis that is worthy of further investigation (Guo et al., 2017; Moses et al., 2020).

### 3.2 Magnetic hyperthermia

**FIGURE 6:**
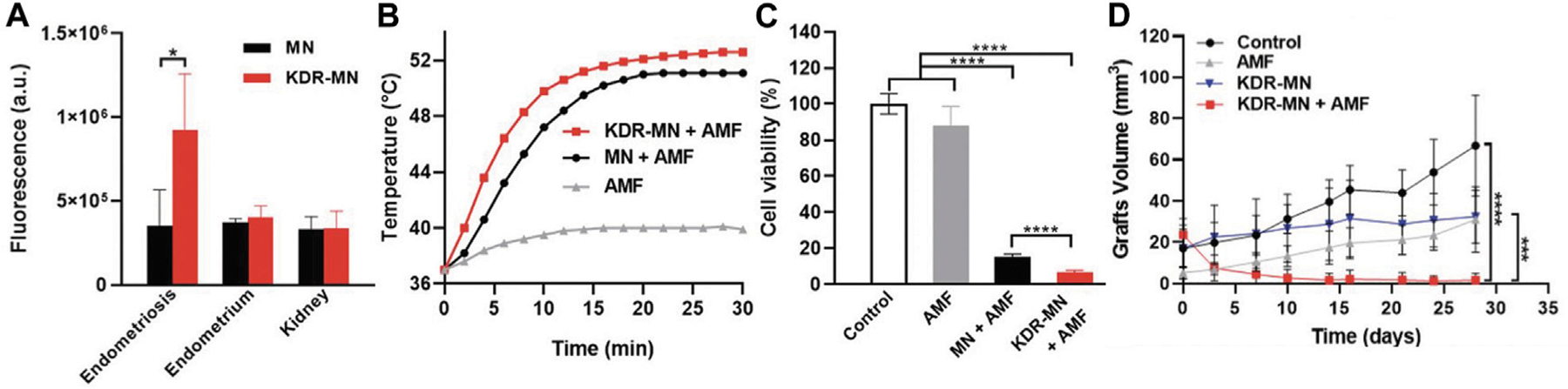
Magnetic hyperthermia of endometriosis mediated by KDR-targeted magnetic nanoparticles (KDR-MN). (**A**) Flow cytometry analysis of cellular uptake (mean ± standard deviation) of dye-labeled non-targeted magnetic nanoparticles (MN, black bar) and KDR-MNs (red bar) by macaque endometriosis stroma cells, endometrium cells, and kidney cells after 24-h incubation. *p < 0.05 by unpaired t-test (n = 5). (**B**) Temperature profiles of endometriosis cells treated with only applied magnetic field (AMF, gray curve), non-targeted MNs + AMF (black curve), or KDR-MNs + AMF (red curve). Nanoparticles were applied for 24 h at 25 μg Fe/mL and AMF was applied 30 min at 420 kHz (26.9 kA/m). (**C**) Viability of endometriosis cells after the treatments applied in (**B**). Data are means ± standard deviation. Statistical comparisons were performed with one-way ANOVA (*n* = 20, ****p < 0.0001). (**D**) Graft volume *versus* time in mice treated with saline (control, *n* = 4), AMF only (*n* = 4), KDR-MN only (*n* = 4), or KDR-MN + AMF (*n* = 5). Data are means ± standard deviation and a one-way ANOVA was used for statistical analysis (***p < 0.001, ****p < 0.0001). Reproduced with permission from Park Y, *et al. Small*, 2022; 18 (24): 2107808, Copyright 2022, Wiley VCH.

While PTT has shown promise in endometriosis management, it suffers from the limited tissue penetration of NIR light, which is on the order of less than a few centimeters. An alternative approach without these limits is magnetic hyperthermia, which uses an alternating magnetic field (AMF) to activate magnetic nanoparticles and thereby produce heat (Park et al., 2022). Park et al. developed hexagonal iron oxide nanoparticles doped with a small amount of cobalt for improved heating efficiency and encapsulated these NPs in poly(ethylene glycol)-block-poly(ε-caprolactone) (PEG-PCL)-based nanocarriers that were further modified with peptides to target VEGFR-2 (also known as KDR) to enable targeted magnetic hyperthermia of endometriosis (Park et al., 2022). They chose to target VEGFR-2/KDR because its expression is elevated in endometriosis lesions when compared to the eutopic endometrium. Both *in vitro* and *in vivo* experiments were conducted to evaluate the potency of the KDR-targeted magnetic nanoparticles (MNs). When primary cells isolated from macaque endometriosis lesions (which were confirmed to overexpress KDR by qPCR) were incubated with the non-targeted or KDR-targeted MNs, those exposed to KDR-MNs heated more rapidly in the presence of AMF owing to improved cellular binding (Figures 6A, B). In turn, the magnetic hyperthermia mediated by targeted MNs more potently suppressed endometriosis cell viability (Figure 6C). This efficacy was maintained *in vivo*, as mice with endometriosis xenografts that were treated with KDR-targeted MNs and AMF exhibited intralesion temperatures above 50°C during treatment. This heating completely eradicated the lesions (Figure 6D), which did not respond to AMF or KDR-MNs alone (Park et al., 2022). While this study did not directly compare graft volume following treatment with non-targeted MNs *versus* KDR-targeted MNs, it suggests that targeted magnetic hyperthermia could provide a safe, non-surgical approach to eliminate deep-seated endometriosis lesions.

### 3.3 Drug-based therapy

Although there are drugs currently used to assist with management of endometriosis, none are curative and they lack stability and specificity. Oxidative stress, angiogenesis, and extracellular matrix degradation are characteristics of endometriosis, so nanoparticle-based drug carriers that attack these underlying mechanisms of the disease could provide better outcomes. For example, nanoparticles could be used to improve the delivery of compounds with antioxidant or antiangiogenic properties or drugs that can inhibit matrix metalloproteinases (MMPs) (Friend, 2017). Promising drug candidates include epigallocatechin gallate (EGCG) and doxycycline, which have antioxidant and antiangiogenic capabilities and can inhibit MMPs, but which lack the stability necessary for clinical use and thus could benefit from a nanocarrier (Yuxue et al., 2023). Towards this goal, Singh et al. loaded both EGCG and doxycycline in poly (lactic-co-glycolic) acid (PLGA) nanoparticles (Singh et al., 2015). When tested in mice with endometriosis induced by intraperitoneal injection of donor mouse uterine tissue, the dual drug-loaded nanoparticles outperformed nanoparticles loaded with either agent individually. In addition to reducing lesion size and number, the EGCG/doxycycline-loaded nanoparticles reduced MMP activity, oxidative stress, and angiogenesis in the lesions, indicating the drugs were working through the expected mechanism of action. These exciting results demonstrate the promise of PLGA nanoparticles to improve drug therapy for endometriosis, although the study did not provide direct comparison against drugs that were not encapsulated in nanoparticles. In separate work, PLGA nanoparticles have been loaded with copaiba oleoresin (CPO), a natural product derived from *Copaifera landgroffii* with anti-inflammatory capabilities (de Almeida et al., 2016). *In vitro*, the CPO-loaded nanoparticles reduced endometrial stromal cell viability, while unloaded nanoparticles had no toxic effects. This reiterates that PLGA nanocarriers appear to be a safe choice of vehicle for drug delivery to combat endometriosis.

As noted above, a key characteristic of endometriosis is oxidative stress, which results in higher concentrations of reactive oxygen species and lipid peroxide markers as the disease progresses. Congruently, the concentration of antioxidants is lower in serum and peritoneal fluids of endometriosis patients (Friend, 2017). To counteract these effects, one could administer antioxidant drugs (either freely or encapsulated in nanoparticles) or nanoparticles that have inherent antioxidant properties. For instance, cerium oxide (CeO_2_) nanoparticles, also known as nanoceria, react catalytically with superoxide and hydrogen peroxide, and have thus been tested in mice with induced endometriosis. The nanoceria not only decreased oxidative stress and inhibited angiogenesis in this model, but also protected against endometriosis-related adverse effects on oocytes, which is critical for successful pregnancy (Chaudhury et al., 2013). Thus, nanoceria are a promising alternative to drug-loaded PLGA for endometriosis management.

While the above examples aimed to use nanomedicine to treat the underlying cause of endometriosis, other nanoparticle designs have been developed for pain management. A-317491 is a selective antagonist of P2X ligand-gated ion channel 3 (P2X3), a receptor in the ERK signaling pathway that has been implicated in endometriosis pain (Ding et al., 2017; Moses et al., 2021). Given its function, researchers loaded A-317491 in nanostructured lipid carriers (NLCs) that were then coated with chitosan oligosaccharide-g-stearic acid (CSOSA) to form CSOSA/NLC/A-317491 (Yuan et al., 2017). Mechanical and heat paw withdrawal tests were used to measure the hyperalgesia of control or endometriosis rats that were previously injected with either saline or CSOCA/NLC/A-317491. The treatment attenuated pain over a period lasting 8–24 h, which was longer than the 2-to-4-h pain relief provided by A-317491 salt. However, whether the effects of the CSOSA/NLC/A-317491 could extend over a longer prior was not evaluated. Nevertheless, this study shows the potential of nanomedicine to alleviate side effects such as pain that are associated with endometriosis.

### 3.4 Gene therapy

Gene therapy is being widely explored as an approach to treat various conditions. In some forms of gene therapy, specific genes are transferred to a set of target cells (via plasmid DNA or messenger RNA (mRNA) delivery) to modify or compensate for a genetic defect (Yuxue et al., 2023). Alternatively, small interfering ribonucleic acid (siRNA) or microRNA molecules can be delivered to silence the expression of overactive genes at the mRNA level. Since nucleic acids cannot be delivered freely owing to their poor stability and unfavourable pharmacokinetic profile, nanocarriers have been utilized as safe and effective delivery vehicles. With respect to endometriosis treatment, the types of nanocarriers that have been tested in gene therapy applications include polymeric nanoparticles, extracellular vehicles (EVs), micelles, and more (Chaichian et al., 2022; Zhao et al., 2012; Wang et al., 2014; Zhao et al., 2016).

In one study, researchers developed stearic acid-grafted chitosan oligosaccharide micelles (CSO-SA) to deliver pigment epithelium-derived factor (PEDF) plasmids to endometriosis lesions (Zhao et al., 2012). PEDF is a protein with anti-angiogenic functions, so plasmids encoding PEDF would be expected to counteract the angiogenic features of endometriosis. In a rat model of peritoneal endometriosis, the CSO-SA/PEDF nanoparticles distributed to endometriotic lesions and, after 2 weeks of treatment, the rats showed a decrease of ~50% in the volume of endometriotic lesions (Zhao et al., 2012). Moreover, there was a significant reduction in microvessel density labelled by von Willebrand factor without a decrease in α-Smooth Muscle Actin-positive mature vessels in rats treated with CSO-SA/PEDF in comparison to controls. These data indicate that PEDF plasmid delivered via CSO-SA carriers can counteract the angiogenesis associated with endometriosis.

In a similar approach, researchers examined the use of polyamidoamine (PAMAM) dendrimers to deliver plasmids encoding endostatin, a potent inhibitor of angiogenesis (Wang et al., 2014). When directly injected into GFP-expressing subcutaneous endometriosis lesions in nude mice, the endostatin plasmid-loaded PAMAM dendrimers significantly reduced lesion size in comparison to control mice based on analysis of GFP fluorescence intensity at days 15, 20, 25 and 30 and by measurement of lesion weight at day 30. The endostatin-loaded PAMAM also reduced microvessel density based on immunohistochemistry analysis (Wang et al., 2014). Future studies that build on this work should evaluate the potency of endostatin plasmids when delivered via dendrimers or other nanocarriers in a systemic, rather than direct, manner, as this would be a more clinically relevant method of administration.

Beyond using plasmid DNA to increase the expression of desired genes, another potential opportunity to advance the treatment of endometriosis is to silence the expression of specific mRNA molecules using siRNA. This approach was explored by Zhao *et al.*, who delivered siRNA against aquaporin 2 (AQP2) using carriers composed of polyethylenimine (PEI) and chitosan oligosaccharide (CSO) (Zhao et al., 2016). Aquaporins (AQPs), transmembrane proteins spread across various tissues, play a crucial role in regulating water transport across cell membranes. Aquaporin 2 (AQP2) is abundantly expressed in endometriosis tissues, and it contains an estrogen-response element in the promoter sequence (Moses et al., 2021). As a result, estrogen stimulation has been shown to significantly increase the migration, invasion, adhesion, and proliferation of immortalized human endometrial adenocarcinoma cells, while AQP2-siRNA significantly reduces these features (Moses et al., 2021). This led Zhao *et al*. to develop PEI-CSO nanoparticles modified with hyaluronic acid (HA), which targets CD44, as AQP2 siRNA delivery vehicles for endometriosis treatment (Yuxue et al., 2023). After 2 weeks of therapy, the size of endometriosis cysts in treated rats was significantly lower in the (CSO-PEI/siRNA)HA group than in the control group. While promising, this study lacked comparison to a control with scrambled/non-targeting siRNA and it did not include analysis of AQP2 mRNA or protein expression to confirm the effect on cyst size was due to the delivered siRNA yielding knockdown of AQP2. Future work should include these controls and experiments to further validate the potential of this approach.

### 3.5 Immunotherapy

**FIGURE 7:**
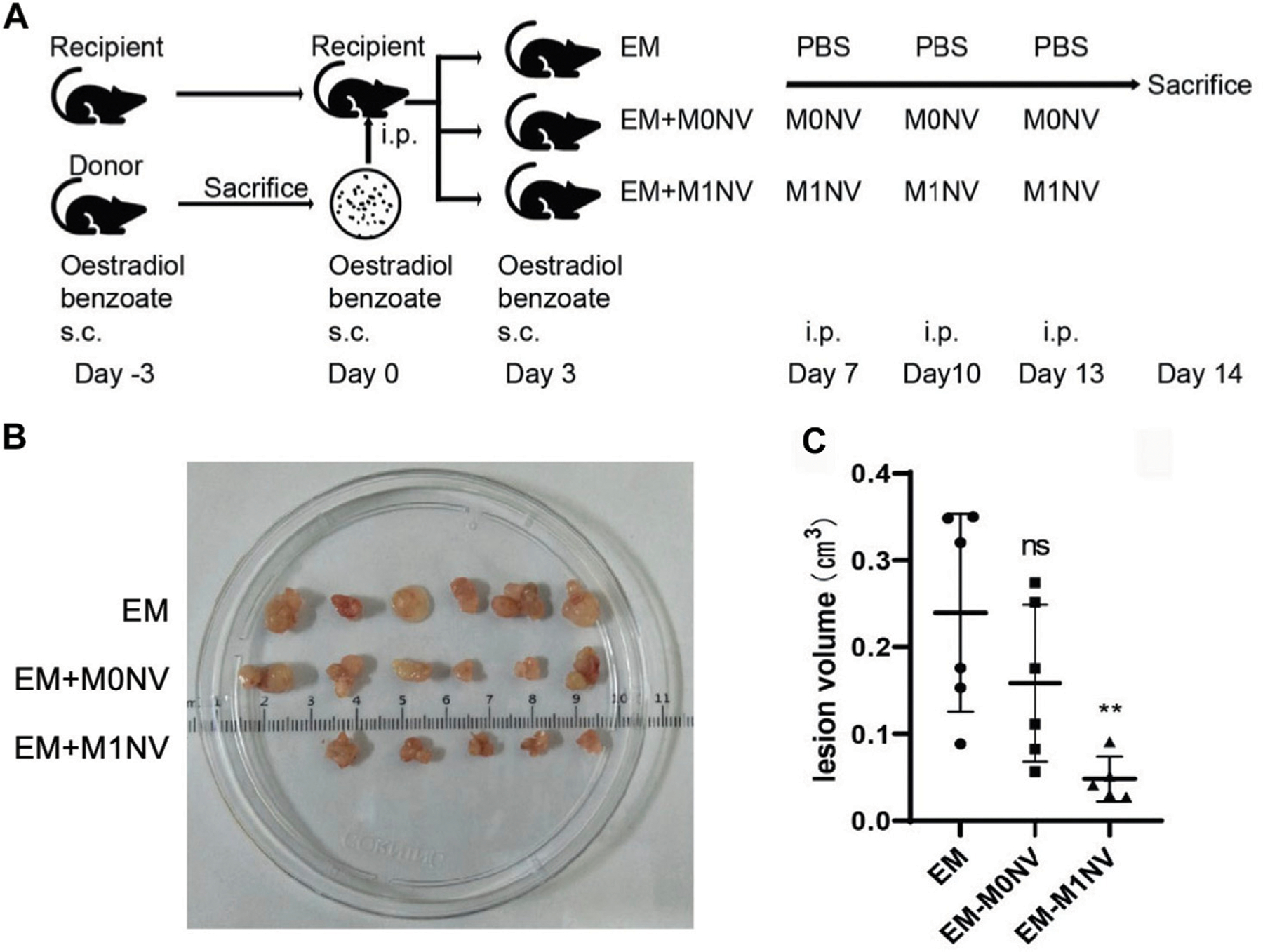
Use of M1NVs to inhibit endometriosis in a mouse model. (**A**) Diagram showing the experimental steps for *in vivo* testing. Oestradiol benzoate was administered to synchronize the cycle 3 days before intraperiteoneal (i.p.) injection of uterus fragments obtained from donor mice. Three days after i.p. injection, mice received oestradiol benzoate again to enhance endometriosis development. Thereafter, they were treated with PBS (EM control), M0NVs, or M1NVs as shown. The dose of NVs was 50 μg/mouse. (**B**) Ectopic lesions excised from mice in each treatment group at day 14. (**C**) The M1NV treatment significantly reduced lesion volume compared to the other treatments (PBS or M0NVs) (ns: no significance when compared with EM, **p < 0.01 when compared with EM). Reproduced with permission from Li Q, *et al. Fronters in Immunology.* 2021; 12: 707784.

Regulating specific immune cells that contribute to disease progression is another promising approach to combat endometriosis. Thus far, studies have focused on macrophages or T cells. Macrophages are of interest because they can recognize and clear endometrial cells from the peritoneal cavity (Moses et al., 2021). The polarization of the macrophages is critical, as a high concentration of M2 macrophages promotes fibrosis and angiogenesis, which contributes to endometriosis development (Li et al., 2021). While transplantation with M1 macrophages has been shown to reduce the growth of endometrial lesions, transplantation with M2 macrophages promotes lesion development (Bacci et al., 2009). Based on this knowledge, Li et al. postulated that M1 macrophage-derived nanovesicles (M1NVs) could reprogram M2 macrophages to the M1 phenotype to suppress endometriosis in a mouse model (Li et al., 2021). Excitingly, the M1NVs reduced the migration of endometrial stromal cells obtained from endometriosis patients *in vitro* and, through the repolarization of M2 macrophages to M1 macrophages, inhibited the disease in a mouse model without any side effects (Figure 7). M0NVs did not elicit this same effect. Although M1NVs have not been investigated further, they show great potential as an option for endometriosis treatment.

Antsiferova et al. conducted another macrophage manipulation-based study (Antsiferova et al., 2013). They evaluated which of two different nanocarriers, unmodified mesoporous silica nanoparticles (UNPs) and aminopropyl modified silica nanoparticles (AMNPs), would be best suited to deliver the immunomodularity drug GMDP (glucosaminyl muramyldipeptide (N-acetylglucosaminyl-N-acetylmuramyl-L-alanyl-D-isoglutamine)) to macrophages collected from the peritoneal fluid of women with endometriosis (Antsiferova et al., 2013). Both particle types demonstrated significant cellular uptake and low cytotoxicity, but when used to deliver GMDP the AMNPs yielded more robust activation of several pattern recognition receptors (including CD36 (scavenger receptor B), CD204 (scavenger receptor A1), NOD2 (nucleotide-binding oligomerization domain 2) receptors, and RAGE (receptor for advanced glycation end products)) in treated peritoneal macrophages compared to free GMDP or GMDP-UNPs. The GMDP-AMNPs also increased expression of MMP-9 (matrix metalloproteinase-9), which helps macrophages degrade the extracellular matrix of cells being targeted for phagocytosis. Unfortunately, this study did not progress into *in vivo* testing, but it will be interesting to see how this technology advances in future work.

T cells are another potential target for immunotherapy of endometriosis. In particular, CD4^+^ CD25^+^ regulatory T cells (Tregs) are directly involved in the upkeep of the body’s immune system. Multiple studies have shown there is an increased concentration of CD4^+^ CD25^+^ Tregs in the peritoneal fluid (PF) of women with endometriosis. Liu et al. investigated the potential for PLGA nanoparticles loaded with anti-CTLA-4-antibodies to inhibit the progression of endometriosis in a murine model (Liu et al., 2017). Blocking CTLA-4 with antibodies can limit the activation of CD4^+^ CD25^+^ Tregs. Using flow cytometry, the percentage of CD4^+^ CD25^+^ Tregs in PF treated with anti-CTLA-4/PLGA or free anti-CTLA-4 was measured. At day 3, there was no significant difference between groups, but at days 7 and 14 there were significantly fewer CD4^+^ CD25^+^ Tregs in samples treated with the nanocarriers compared to the free antibodies. In follow-up studies, ectopic endometrial cells (EEC) were co-cultured with CD4^+^ CD25^+^ Tregs isolated from PF and exposed to anti-CTLA-4/PLGA or free anti-CTLA-4. EEC proliferation and invasion were measured via ELISA and Matrigel invasion assays, respectively. The antibody nanocarrier treatment significantly suppressed EEC proliferation and invasion, and the effect was more pronounced than that of free antibodies, which the authors attributed to the sustained release from the PLGA (Liu et al., 2017). These results, coupled with observed reductions in the expression of IL-10 and TGF-beta cytokines, suggest anti-CTLA-4/PLGA could be a promising nanomedicine for endometriosis therapy.

## 4 Discussion/future outlook

As there is no cure for endometriosis and a paucity of treatments with long-lasting effects, it is essential to develop novel approaches to combat the disease. Further, too many women go undiagnosed for years due to a lack of accurate and simple diagnostic tools, and it is even more challenging to characterize the severity of the disease. These unmet needs have recently led to an influx of researchers working to develop improved treatment and diagnostic options for endometriosis, including approaches based on nanotechnology. Table 1 summarizes the technologies discussed in this review. Nanoparticles have numerous advantages in that many are biocompatible and can be easily modified with desired cargo, targeting ligands, or imaging agents. This has led to a great deal of progress towards developing non-invasive, accurate diagnostic tools and safe, effective treatments for endometriosis. Indeed, as discussed in this review, new treatments based on hyperthermia, drug delivery, gene therapy, and immunotherapy have shown substantial promise thanks to the unique characteristics of nanomedicines. Likewise, new sensors and imaging agents have shown promise to support earlier detection, which would transform patient care.

Despite these exciting advances, there is still work to be done. Regarding biosensors, there are many potential non-invasive biomarkers that require more testing to validate their use in endometriosis diagnostics, especially miRNA and siRNA, which are underexplored (Zhang et al., 2020). With respect to imaging, although several techniques have been tested for cervical cancers and other reproductive/gynecologic diseases, their application to endometriosis remains to be investigated. Approaches including fluorescence imaging, photoacoustic imaging, computed tomography, and multimodal imaging have great promise to advance the detection and delineation of endometriosis (Luo et al., 2023a). Future work incorporating nanoparticles, particularly those targeted to specific biomolecules on endometriotic lesions, as contrast agents could greatly improve diagnostic imaging and staging of the disease. Moreover, systems that provide both imaging and treatment (such as those discussed that enable imaging and PTT) could greatly advance patient care and outcomes (Guo et al., 2017; Moses et al., 2020).

Future steps for treatment should include both innovations in the nanotherapy design and the development and incorporation of improved and more accurate animal models of the disease. Current animal models usually utilize mice and rats, but they do not develop endometriosis naturally and the pathogenesis of the disease is complex. A primate model would be better suited for testing endometriosis treatments as the primate reproductive system has more direct similarities to that of humans and researchers would be able to naturally induce endometriosis. In addition to validating nanomedicines for endometriosis in more accurate disease models, future work should test nanomedicines appropriately for safety, which is crucial to ensure their translatability to humans.

Beyond typical safety analyses that measure blood chemistry, serum cytokines, body weight, and other characteristics of treated animals, future studies should ensure treatments do not negatively impact fertility, as infertility is a major complication of endometriosis. There is still much to be learned surrounding the mechanisms of endometriosis-associated infertility, so initial steps will require these mechanisms be determined, and then interventions established. Women with endometriosis have increased levels of peritoneal fluid and IL-6 levels are increased in the PF; together, these decrease sperm motility by 40%–80% and also have other detrimental effects on embryo development and sperm (Oral et al., 1996; Harada et al., 2002). Given this knowledge, ensuring treatments can induce/maintain a healthy pelvic environment may be a critical requirement for reducing the infertility of endometriosis.

In conclusion, researchers are actively developing various diagnostic and therapeutic tools based on nanotechnologies which may someday improve the quality of life and care for endometriosis patients. Until now, the application of nanomedicine to women’s health has been understudied. More research is needed for these technologies to realize their potential, but it will be immensely exciting to follow the field as it progresses and transforms the lives of women with endometriosis.

## Figures and Tables

**TABLE 1:** Summary of the nanotechnology-enabled diagnostics and treatments for endometriosis presented in this review.

	System components	Application	Key results	Citation
Biosensing	Multiwalled carbon nanotubes and Fe_3_O_4_ NPs dispersed in chitosan, coupled to anti-CA19–9 antibodies, and placed on glassy carbon electrodes	Detection of CA19–9 in serum via electrochemical immunosensing	Detectable range of 0.00083 to 83 IU/mL	Kalyani et al. (2021)
	Glassy carbon electrodes modified with gold NPs, reduced graphene oxide, and CA-125 antibodies	Detection of CA-125 in blood via electrochemical immunosensing	Detectable range of 0.0001 to 300 IU/mL	Sangili et al. (2020)
Imaging	Fe_3_O_4_ NPs coated with hyaluronic acid (HA) to target CD44 on endometriotic cells	Lesion visualization in a rat endometriosis model via FS-T2-weighted MRI	Significant MRI contrast enhancement of lesions by 2 h post-IV injection of NPs	Zhang et al. (2014)
	Ultrasmall superparamagnetic	MRI detection of intraperitoneal endometriosis lesions in a rat model	Successful detection of lesions >3 mm in	Lee et al. (2012)
	Fe_3_O_4_ NPs			
	Hexagonal Fe_3_O_4_ NPs coated with peptides targeting VEGFR-2	MRI and fluorescence imaging of lesions in mice bearing transplants of macaque endometriotic tissue	IV-administered NPs enhanced lesion contrast under both imaging modalities	Park et al. (2022)
	SiNc-PEG-PCL NPs (silicon naphthalocyanine loaded poly (ethylene glycol)-poly(ε-caprolactone) NPs)	Fluorescence imaging of endometriotic grafts in a murine model	Significant fluorescence observed in grafts 24 h post-IV injection of NPs using an FDA-approved intraoperative imaging system	Moses et al. (2020)
Therapy	SiNc-PEG-PCL NPs	PTT of endometriotic lesions in mice upon NP activation with 780 nm light	PTT yielded >95% cell death *in vitro* and complete disease eradication without recurrence over 7-week *in vivo*	Moses et al. (2020)
	TNYL-HAuNS (Hollow gold nanoshells modified with TNYL peptides that bind EphB4 receptors)	PTT of endometriosis in mice upon NP activation with 780 nm light	TNYL-HAuNS exhibited ~2-fold higher accumulation in lesions than non-targeted HAuNS; PTT inhibited lesion volume by 92.7%	Guo et al. (2017)
	Cobalt-doped hexagonal Fe_3_O_4_ NPs loaded in PEG-PCL carriers modified with peptides targeting VEGFR-2	Magnetic hyperthermia of endometriosis xenografts in mice	Complete eradication of lesions achieved *in vivo*	Park et al. (2022)
	Epigallocatechin gallate (EGCG) + Doxycycline loaded in PLGA NPs	Drug-based treatment of intraperitoneal endometriosis in a murine model	Treatment reduced lesion size and number and also decreased MMP activity, oxidative stress, and angiogenesis in lesions	Singh et al. (2015)
	Copaiba oleoresin (CPO)-loaded	Anti-inflammatory drug-based therapy	Reduced endometrial stromal cell viability *in vitro*	de Almeida et al. (2016)
	PLGA NPs			
	CeO_2_ NPs	Management of endometriosis in a murine model through the antioxidant properties of the NPs	Treatment decreased oxidative stress, inhibited angiogenesis, and protected against adverse effects on oocytes	Chaudhury et al. (2013)
	A-317491 loaded in nanostructured lipid carriers coated with chitosan oligosaccharide-g-stearic acid (CSOSA/NLC/A-317491)	Pain management in a rat model of endometriosis	Treatment attenuated pain over 8–24 h, compared to 2–4-h relief provided by A-317491 salt	Yuan et al. (2017)
	CSO-SA micelles loaded with PEDF plasmids	Anti-angiogenesis gene therapy of endometriosis in a rat model	Treatment decreased lesion volume by ~50% and significantly reduced microvessel density	Zhao et al. (2012)
	PAMAM dendrimers carrying endostatin plasmids	Anti-angiogenesis gene therapy of endometriosis in a subcutaneous murine model	NPs directly injected into lesions reduced lesion size at days 15, 20, 25, and 30 and decreased microvessel density	Wang et al. (2014)
	CSO-PEI nanocarriers coated with HA and carrying AQP2 siRNA	Gene regulation of endometriosis in a rat model	Endometriosis cysts were significantly smaller in (CSO-PEI/siRNA)HA-treated rats after 2 weeks of therapy	Yuxue et al. (2023)
	M1NVs (M1 macrophage-derived nanovesicles)	Immunotherapy of endometriosis in a mouse model by reprogramming M2 macrophages to an M1 phenotype	Successfully inhibited disease *in vivo* without any side effects	Li et al. (2021)
	Unmodified mesoporous silica NPs (UNPs) and aminopropyl modified silica NPs (AMNPs) loaded with the immunomodulatory drug GMDP	Immunotherapy *in vitro* using macrophages collected from the peritoneal fluid of women with endometriosis	Both NPs exhibited significant cellular uptake and low cytotoxicity, but GMDP-AMNPs increased immune activation	Antsiferova et al. (2013)
	PLGA NPs loaded with anti-CTLA-4 antibodies	Endometriosis management through immunotherapy to limit the activation of CD4+ CD25+ Tregs	Treatment reduced percentage of Tregs in peritoneal fluid and decreased endometrial cell proliferation and invasion	Liu et al. (2017)
